# Fulton County Medical Society, Regular Meeting, November 20, 1913

**Published:** 1914-01

**Authors:** R. R. Daly


### FULTON COUNTY MEDICAL SOCIETY.

### Regular Meeting, November 20, 1913.

By R. R. Daly, M. D.

Dr. Robin Adair read a paper "Dangers from Abscessed Teeth."

Dr. Daly said that in his practise it frequently happened that patients complained of pharyngitis and tonsillar troubles which were due to the infection coming from diseased and abscessed teeth. He referred such cases to the dentists so that the cause could be removed.

Dr. Rhodes said that he had a patient operated upon for cataract recently and the subsequent symptom was great pain in the jaw. It was feared that the operation had gone wrong in some way, but a careful examination found a coincident abscess forming around a snag of a root under a plate. The removal of this relieved the pain and permitted an uneventful recovery.

Dr. Niles said that he had had a striking experience in his own family with difficulties of this kind. His own child had suffered since he was thirteen months old with recurring attacks of fulminating fever where the temperature jumped suddenly to 105 and even to 107. He consulted his professional friends and everything they could think of was done from time to time, but the attacks recurred and he was in great fear of the child's life each time. There was evidently some source of toxic infection that could not be located. As the child grew older and could make himself better understood, it was learned that there was tenderness in the teeth and Dr. Adair was consulted. Four teeth were removed and each was abscessed at the root. The attacks of fever ceased for a long time but there had been a mild one recently and the child was again under Adair's observation.

Dr. Adair said in closing that Niles' case was a sad one because there was no possible way by which drainage of these abscesses could be accomplished except by taking the teeth out. He tried lateral methods without success. He doesn't know what effect the removal of the teeth at this early age will have upon the development of the jaws, but the operation had to be done to save the child's life.

The cases he had described in his paper were not sensational ones, he said, but those occurring frequently and likely to be overlooked by the general practitioner. Teeth should be tapped by any hard substance whenever there was the least reason to suspect mouth difficulty and whenever there was any tenderness found, it should be investigated.

Dr. W. A. Gardner read a paper on "Hereditary in Nervous Diseases and its Social Bearing."

Dr. Niles in discussing the paper, related an incident in the career of the late Editor Brand of the "Iconoclast.". It seems in discussing a solution of the race problem, some man suggested that the best solution was to "cross it out". This made Brand highly indignant and he expressed himself in the following words:

"Any man so vile as to suggest the solution of the race problem, by the miscegenation of the Caucasian with the Ethiope is not worthy to die a decent death. He should be bitten by a blue-gum nigger, clawed by a buzzard, kicked to death by a blind jackass and buried face downward in a pile of compost."

No matter what the hereditary of disease, it was not to be crossed out without the utmost care in the selection of the parents.

Dr. W. N. Adkins read a paper "Diagnosis of Scarlet Fever".

Dr. Boynton said that scarlet-fever has caused him more trouble than any other disease. He isolated his own boy for three weeks and he never peeled, he is not sure whether the rash was scarlet-fever or not. He has a case now that looks like it, but it is so atypical that he doesn't know what to do about isolation. He had a case several years ago in which there were six weeks' isolation followed by a renewal of the rash two weeks after freedom from quarantine. Dr. Kennedy, of the Health Board, saw the case and confirmed it at that time. This was the shortest interval between attacks he had ever heard of. The second attack was typical and lasted four weeks.

Dr. L. B. Clarke said that Dr. Adkins' description of the disease was perfect but that he did not altogether agree with him as to its course. It is a great pity that the pathologists have not discovered the germ so that we can be definite about it and tell whether the borderland cases are to be considered as true scarletina. He has long believed that it is a modified streptococcic infection but can offer nothing but the clinical symptoms to support the idea. He has seen a case under one year of age. There were five cases in the family and a nursing baby, eight months old had it with the others.

The rash never begins on the face. This is important to remember.

Dr. Varden said that cervical enlargements are common in children anyhow and should not have much importance attached to them in this disease.

Dr. Duncan called attention to the rapidity of development after invasion and told of visiting a family where there were two little children. The elder had the fever, the other seemed all right and he took pains to observe it. On the third visit to the house the younger was moribund and died in a few hours.

Dr. M. T. Davis asked if it was in the experience of the others that strawberry tongue was present in some epidemics and absent at other times. He had thirty cases in one group and the strawberry tongue was marked in each case.

Dr. Adkins said in closing that he never disputes any man as to what that man has seen. Cases thus may have died under a year of this trouble. As to reinfection, he has heard of cases who claimed it but has never seen it. An Italian man once presented himself at the hospital who appeared at first to have a recurrent case of scarlet-fever but it was hemorrhagic smallpox. Just by chance it was noted and the man was not put in a scarlet-fever ward.

So far as the face is concerned, he never has seen the rash there at any time. There is a flush with the fever and there may be pimples that are aggravated by the condition, but there is no real rash. He agrees with Varden as to the relative importance of cervical adenitis, but no one symptom is enough to settle the diagnosis. The glands are always enlarged when the disease is present and has been there for three days, including the prodromals. He has seen several nursing children under a year left with their scarlet-fever mothers, but none took the fever. As to the tongue. All the cases he has seen have been in the hospital. Most cases have it at sometime usually after the rash. He understands strawberry tongue to mean a clean, red tongue with the papillae standing up.

MEETING OF THE SOCIETY, DECEMBER 4, 1913.

Dr. Daly reported a case of deflected septum at birth. No instruments have been used, but the nose was bent over on the face and the cartillage had grown in that position for a time long enough to make it resume that angle when lifted to the normal. The nose was splinted internally and the position thus maintained. The child was two days old when this was done.

Dr. L. Amster gave "Lantern Slide Demonstrations of X-ray diagnosis of Diseases of the Stomach."

First, he told of his personal experience and how for years he had suffered with gastric symptoms that simulated ulcer. There had been hyperacidity, vomiting, pulling sensations in the stomach at night, depression and the like. He was told that he had neurasthenia and that he must quit work and go abroad. He had been doing that for several summers but with nothing more than temporary benefit. He was examined in Frankfort and the X-ray showed an intense peristalis. There were many examinations with the same result—neurasthénia.

One man here, Dr. McRae, had persistently told him that he had appendicitis, but he could not be convinced. He returned last September from Berlin and had a bad attack in New York. He was examined and the diagnosis of ulcer of the stomach was made and operation occurred. The stomach was found to be all right but an extension of the operation disclosed an appendix with two strictures and a calcareous half. The removal of this was followed by complete relief from all the symptoms, an increase in weight and a restoration of ability to work hard. It is a lesson in careful analysis in making diagnosis in all abdominal cases. An exploratory operation should never be withheld.

Dr. E. G. Jones said that he was glad this case had been reported because many cases of indigestion are not stomach troubles at all.

Dr. Cartlege said that the Mayos had reported that 95% of the cases complaining of stomach trouble in their clinic were not gastric at all.

Dr. Niles said that Dr. Amster's case was interesting. He has seen several cases where the trouble was not in the stomach, but in the appendix and it is becoming realized that there are many cases of apparent gastric difficulty that have remote causes. These are being searched out and knowledge of the reflexes is increasing. But still there is a word to be said for the internist and after these Goliaths have had their say, he as an humble David would essay the promulgation of the fact that in the search among other organs the stomach may be forgotten. It is still there nevertheless and it is not always doing its work well. When the X-ray shows certain gastric symptoms in accord with clinical conditions, something must be done and the fight is on. Othello feared his occupation was gone at one period of his career. That was war. And as to stomach diseases Niles is still in the fight.

Dr. S. R. Roberts read a paper "Treatment of High Pressure Diseases".

Dr. A. B. Elkin said that he was greatly interested in this subject because he has been trying to account for pressure of 220 at age of 36. He has never used aconite as suggested because he is afraid of it. Has used liquid diet, rest and veratrum, and most cases improved.

Dr. Lokey said that he is in the habit of taking the blood pressure in all elderly patients of failing eyesight whether there are symptoms of glaucoma or not. It forms a good guide in further treatment and is of especial value in tnnitus and vertigo.

Dr. Crenshaw called attention to the relative amount of liquid intake and counselled that it be kept low in increased pressure. He has used F. E. Phytolacca successfully and it tends also to reduce obesity. He asked as to the effect of very hot baths and seemed to think that there was increase of pressure when they were used.

Dr. Thrash said that we are likely to get mixed in the name of the disease when we call it High Pressure Disease. It is really merely a symptom. Capillary Arterio Sclerosis is better. The capillaries are the first to suffer. Only the intima forms their walls and when they are inflamed this turns to fibrous and inelastic tissue. It prevents free passage of blood along the lumen and interferes almost completely with the endosmosis. The strain works backward to the larger vessels and vascular crises occur. There are pains in various parts of the body other than the heart and thus we have the disease.

Dr. Blosser called attention to the abstinence from food of high lime values as a cure for this disease.

Dr. Duncan said that he was not afraid to use either aconite or veratrum. They and the nitrites are safe if we know how to use them. He has high blood pressure himself and he doesn't bother about it. He thinks it a provision of nature.

Dr. Kime said the subject is a forerunner of what the physician of the future will have to consider. We live in a high pressure age and we do things under unnecessary stress. The heavy, indigestible foods, coffee, alcohol, tobacco, etc. all tend to increase pressure and to induce it. Primitive man with his very active life was able to eliminate the products of the coarse and heavy food he used, but we are not. The average American is a hot house plant and he must change his habits if he will remain well. Veratrum and aconite should be carefully watched. Veratrum is safer and may be administered two minims t.i.d. for a long time.

Dr. G. W. Quillian said he has had recent unhappy experience with high pressure. His patient, a prominent clergyman had pressure of 212 last March which was reduced to 165 by rest and small doses of sodium nitrite. The gentleman returned to active work against the doctor's advice and persisted in it till pressure was 218. Aconite was then given and the return to rest strongly urged. The advice was not taken and the patient fell dead in the pulpit a short time ago.

Dr. Funke said that the title had mislead him and that he came expecting to hear something about Caisson Disease.

Arterio-capillary Fibrosis is due to change in the arterioles. The kidneys do not show changes in the capillaries of the tufts, but the capsules and these gradually impinge upon the tufts, but in the capsules and these gradually impinge upon intimal structures in the Aorta show it early because they get the least quantity of blood. He cannot agree that with failing heart there is increased blood pressure. Apoplexy doesn't occur so frequently in degeneration of the myocardia. High tension is in a sense a physiologic compensation for the changes in the vessels.

Dr. Roberts answered saying that he was trying to make a synthesis of a vast number of things and that 15 minutes was a short time. He must admit that there were modifications to some of his statements.

As to syphilis, there is dilated heart aortitis and patches of atheroma in the Circle of Willis. High pressure cases should not bathe hotter than 95 to 100 for the dilation of the vessels would strain the heart.

There is a multitude of things at work to cause this difficulty. Two railroad presidents died in one week of the trouble autopsy showed pus in the lateral sinus.
and they lived diverse lives, of course.

Dr. C. W. Strickler head a paper on "Post Typhoid Sepsis."

Dr. Cartlege said he had never seen the condition described, but it reminded him of a case who died and at the

Dr. Strickler added that this was what the old practitioners were wont to call typho-malarial fever. There was nothing to indicate the condition Dr. Cartlege mentioned.

MEETING JANUARY 15, 1914.

Dr. Dunbar Roy read a paper. "Some Observations on Medical Europe."

Dr. Barnette said that though Spain had not been mentioned in the paper, he was convinced that the conditions in that country were deplorable and that a tourist in search of improvement would go there only to see what the country needed and not to learn advanced methods in the profession of medicine.

Dr. Funke said that there was another center for Americans who were always in Vienna. The Cafe Clinic was a new headquarters and there could be obtained detailed information of all that a man was likely to want to know in his search for clinical work and instruction.

In London, Paris, Berlin, Leipsic and Dresden there is not the convenient and practical system there that is to be found in Vienna. These other cities have fine clinics but they are more scattered and, too, one must as a rule, know the language of the city. Berlin has special courses at certain times of the year and one must know of this in order to get them.

Dr. Roy closed saying that if one wanted to get complete information concerning Vienna matter, he had only to write to the American Association there to secure pamphlets that will cover every question. There is as good teachings to be had in this country and as much clinical material but it is not so well arranged. Moreover, the teachers there give their whole lives to the work and take only practice for a living incidentally. This spirit of devotion to teaching pervades everything and stimulates the student to an unwonted interest in his labors. A man who in New York would set some of his time apart for other things, would, were he in Vienna, never think of matters outside of the subjects he came there to study. The atmosphere is different and one likes the hard work.

Dr. Hansell Crenshaw read a paper "Classification of Mental Diseases".

Dr. Gardner said that there are so many classifications that in practice a man uses the one of his favorite author. He thinks that with the exception of the omission of Paranoia J. B. Luke's classification is the best.

Dr. Brawner said that he attempted a classification of 20 cases in his Sanitarium and found 4 Toxic, 3 Alcoholic, 1 Involutional Melancholia, 3 Manic Depressive, 6 Dementia Præcox, 1 Epilepsy, 1 Paranoia, 1 Senile Dementia. These were practically all etiologic in classification.

Dr. Duncan asked if softening of the brain was senile dementia.

Dr. Crenshaw said that he had overlooked Pellagra in his classification, but that was due in part to the fact that it had no definite form of insanity and that in the hospitals it was added to the dominant mental trouble, for instance "Manic Depressive with Pellagra."

Dr. E. C. Cartlege read a paper "Childbirth, Perineal Tears, Forceps, Chloroform Massage."

Dr. E. C. Davis said that the subject was so comprehensive that he could not undertake to discuss it in one evening. He does not agree in always keeping a woman covered, for he believes he needs the aid of all his senses in doing his work and in preventing infection.

Dr. McCord said he must disagree largely with the essayist. Forceps cannot be applied in almost every case. Good men never use them until they are absolutely necessary. He can not administer chloroform with one hand and deliver with the other and do either of them well.

The way to prevent tears is to keep the head flexed. It can be held back better in this way and then it is ready when delivery comes. But no matter what the method, tears will occur. In over 800 cases he has had some degree of laceration in three out of five cases. As to examining a patient under a sheet, he would note that it is the vulva and not the vagina and uterus that have pyogenic bacteria. This vulva should be not only cleansed, but seen when one is at work if he wants to shorten the puerperal state.

Dr. Donaldson said that obstetrics was a matter of opinion and some quarreling still and that the individual had each his way. The use of forceps depends upon one's familiarity with them and men differ in that. If he knew of some way to prevent tears he would be very happy, because as a lady once said to him in rather a cryptic way that when a tear has occurred "it is never as good afterward." He doesn't insist upon the mechanism in the books as being necessary in every case. He has it as a guide but avoids "meddlesome midwifery" and lets nature take her course till he is sure there is something wrong.

Dr. Kime pointed out that obstetrics vary with the locality. In the city there are steam heated houses and hospitals where the lady can be cared for almost as one likes, but in the country where the temperature may be way below freezing and there is scant fire in the house, one must consider the patient and keep her covered. Chloroform correctly given is safe and good. It must be given just before the height of the pain so as to assist in holding it back when it is too severe. If it is given on a folded handkerchief, it can be handled by the obstetrician with ease and will not interfere with his other work. Hot applications to rigid perinaeum will be of assistance oftimes. Forceps should be so shaped as not to slip on the head. That is the main qualification. A glove on the right hand prevents infection. Sterile dressings can be had in the cotton and gauze at any drug store and they are as efficient as the more expensive ones at the hospitals.

Dr. Duncan said that a man once told him that he had never had a tear. Duncan did not believe him. He likes to see what is going on when he is delivering a woman and spoke of a cord around the neck as an instance when vision was likely to be of some avail. His rule as to forceps is not to prolong the second stage.

Dr. Cartlege said in closing that he meant to discuss just the things in his title and that he had done so. They were all important and men had various opinions about them that were well to discuss. He never meant to say that he would not uncover a woman when he used forceps nor at any other time when it was necessary, but he was convinced that women were less nervous and excited when covered and he kept them protected as long as he could. He never has had infection in his cases. The secret of successful delivery is to go slow.

